# Some Interesting Enquiries, Respecting the Manner in Which Nourishment Is Carried over from the Mother to the Fœtus

**Published:** 1806-03

**Authors:** 

**Affiliations:** Professor of Midwifery, of Strasburg


### Some interesting Enquiries, respecting the Manner in which Nourishment is carried over from the Mother to the Fœtus. *By* Dr. Lobstein, *Professor of Midwifery, of Strasburg.*

According to Dr. L. the liquor amnii is the principal source of nourishment to the fœtus. In all warm or cold blooded animals, even in those where the placenta or the umbilical cord is wanting, we find the fœtus swimming in this liquor. Towards the period of birth it decreases, and we observe that this liquor never undergoes any kind of corruption, though it assumes sometimes a more deeply saturated yellow colour. As it cannot be absorbed by the membranes surrounding the fœtus, for want of lymphatic vessels, it follows, that it must be taken up gradually by the child itself, and by the exhaling vessels of the skin be again reproduced. Moreover, as in all kind of animals, when the fœtus has no umbilical string, there can be no other way of nourishment, why may this not be the same in the human animal? According to the chemical analysis of Vauquelin and Buniva, this liquor contains albuminous particles, though not in a large quantity; yet always in greater proportion during the first months of pregnancy. The Doctor refutes the opinion that the liquor amnii is swallowed by the child; on the contrary, he asserts that it is taken up by the absorbents on the surface of the body only. Another source of nourishment is the *placenta*. By a minute enquiry it is found that the veins exist sooner than the arteries; consequently, they must perform other functions than carrying the blood back from the arteries. It is probable that they absorb at the same time, if not in every part, at least in some. The Doctor is therefore inclined to assert, that in the first period of pregnancy, the veins existing on the chorion, and the placenta without arteries, may answer the purposes of absorbents. In the first months of pregnancy, we observe between the uterus and the placenta a milky liquor, like chyle. It is therefore probable that the veins on the membrana caduca take up this liquor, till the umbilical arteries are formed, and the placenta is more perfect, when the anastomoses of the arteries and veins take place. The third way by which nourishment is carried to the fœtus is the *allantois*; probably the lymphatics of the urinary bladder of the fœtus carry the nourishing liquor of the allantois. The fourth source of nourishment is *the umbilical cord*. It increases im in length as pregnancy goes on, and the albuminous matter contained in its cells may be carried to the child, and be absorbed by the lymphatics of the peritonæum. In quadrupeds, where the umbilical cord is very short, we find the whole surface of it surrounded with little vesiculæ, which renders this opinion still more probable.

